# Effect of citric acid on phytoextraction potential of *Cucurbita pepo*, *Lagenaria siceraria,* and *Raphanus sativus* plants exposed to multi-metal stress

**DOI:** 10.1038/s41598-023-40233-2

**Published:** 2023-08-11

**Authors:** Ehab A. Ibrahim

**Affiliations:** Vegetables Research Department, Horticulture Research Institute, Agricultural Research Center, 9 Cairo University St., Orman, Giza, Egypt

**Keywords:** Pollution remediation, Environmental impact

## Abstract

Phytoextraction is a novel technique that involves using plants to remove heavy metals from contaminated soils. An outdoor pot experiment was designed to evaluate the phytoextraction potential of three plant species *Cucurbita pepo, Lagenaria siceraria*, and *Raphanus sativus* in soil contaminated with multiple metals (Cd, Co, Cr, Cu, Ni, Pb, and Zn) under the application of citric acid. The results showed that *Raphanus sativus*, out of all the studied plants, had the highest root and shoot dry weight and the capacity to accumulate all heavy metals at higher concentrations except for Cu. The application of citric acid into the polluted soil significantly increased plant growth, biomass, and heavy metal uptake. High bioconcentration values indicate that *Raphanus sativus* is a promising plant for absorbing and accumulating Cd and Ni from the soil. The maximum values of bioconcentration were also observed by the application of citric acid. The values of metal translocation from the root to the shoot were varied by plant species and the citric acid application. Regarding the biomass, metal content, as well as removal metal percentage values, it became apparent that the *Raphanus sativus* plant was the most effective crop in removing heavy metals from multi-metal contaminated Soil. Generally, these findings emphasize that the application of citric acid could be a useful approach to assist Cd and Ni phytoextraction by *Raphanus sativus* plants. When these plants are growing as vegetable crops, more attention should be given to evaluating the heavy metal content in them, especially when adding citric acid to their soil through fertigation systems to avoid food chain contamination.

## Introduction

Heavy metals have contaminated agricultural soils in many parts of the world. This is due to modern agricultural practices. Various agricultural activities like the use of organic and inorganic fertilizers, the application of excessive amounts of pesticides, and irrigation with low-quality water have been considered as main sources of heavy metal contamination in agricultural soils^1,2^. The accumulation of heavy metals in agricultural soil can result in degrading the quality of the soil and groundwater quality^3^. These can threaten crop productivity and human health throughout the food chain^4,5^. Thus, it is critical to remediate heavy metals in agricultural soils. Various approaches have been developed for resolving this environmental issue^6^.

The use of specialized and highly adapted plants to absorb, transport, and accumulate heavy metals in the biomass of harvestable organs from contaminated soil is known as phytoextraction^7,8^. Phytoextraction has recently gained popularity due to its cost-effectiveness and environmentally beneficial nature^6^. However, the efficiency of uptake and translocation of heavy metals to harvestable parts may differ depending on plant species, soil type, and environmental conditions^9–11^.

Although there are 400 species of metal hyperaccumulators (species with the capacity to accumulate significant amounts of metals from the surrounding soil in their aerial tissues) and they have been widely studied for metal phytoextraction, their use for phytoextraction of metal-polluted soils presents challenges because of their small size, low biomass production, and lack of any well-established cultivation, pest management, or other management practices^12,13^. To overcome these restrictions, there is a lot of interest in finding and developing fast-growing, high biomass hyperaccumulators, tolerance to high pH and salt, and resistance to diseases and pests as well as investigating and implementing better agronomic practices to improve phytoremediation efficacy^12,14^.

Plants were divided into three categories based on how well they could absorb heavy metals^15^: low accumulation (e.g., *Leguminosae*), medium accumulation (e.g., *Cucurbitaceae*), and high accumulation (e.g., *Brassicaceae*). The *Brassicaceae* family has the most hyperaccumulator species, accounting for roughly a quarter of all known hyperaccumulators^16,17^. The phytoremediation capacity of various *Brassicaceae* plants such as radish (*Raphanus sativus* L.) has been extensively investigated. Because radish is a hyperaccumulator plant that can concentrate heavy metals in its various parts, it is useful for the remediation of contaminated areas^18–20^. Metal extraction from the soil by radish occurs up to a certain concentration, after which the phytoextraction rate of metal or bioaccumulation coefficient decreases as the concentration of metal increases^19^. Because radish may be sown up to five times per year and produce up to 20 t ha^−1^, it can be used to remediate lead-polluted topsoil (0–10 cm)^18^. Although phytoextraction studies with radish have achieved promising results in mono-metal soils, the synergistic effects of this plant with chelating ligands in multi-metal settings have received far less study.

Certain plants, especially those belonging to the *Cucurbitaceae* family such as summer squash (*Cucurbita pepo* L.) and bottle gourd (*Lagenaria siceraria*) can take up and accumulate significant amounts of heavy metals from contaminated soil. Summer squash has been examined as a potential phytoremediator for the removal of Co, Cr, Cd, Zn, Cu, Ni, Pb and Mn from polluted soils^21^, and it could accumulate Co, Cr, Zn, Cu, Ni and Pb into leaves and roots^22,23^. Moreover, Eissa^24^ and Ibrahim et al.^25^ studied the uptake and translocation of heavy metals in summer squash and stated that heavy metals were significantly accumulated in roots and shoots. Bottle gourd plants can absorb heavy metals through their roots and transfer them to the plant's shoot^26^. However, there is insufficient evidence of the behavior of these plants in multi-metal contaminated soils.

Unfortunately, some limitations limit the applicability of phytoextraction, such as heavy metal concentration levels in the soil cannot be too high due to their toxicity, variations in soil pH affect the bioavailability of metal ions, remediation occurs predominantly in the rhizosphere at shallow depths, limited knowledge of plant behavior and phytoextraction capacity, and difficulty in managing plant crops^9,11^.

Generally, poor HM bioavailability and mobility of heavy metals within the rhizosphere limit natural phytoextraction efficiency, which is influenced by soil pH and clay content, plant tolerance to HMs stress, soil nutrient levels, and HM selectivity^8,27^. Heavy metal dissolution in the soil is mostly influenced by pH^28,29^. Because of the limited solubility, high pH (alkalinity) can reduce metal bioavailability^30^.

As a result, chemically assisted phytoextraction (also known as chelate-enhanced phytoextraction) has been used to boost HM solubility and bioavailability^6,8,31^. Ethylenediaminetetraacetic acid (EDTA) is one of the most effective chelating agents and has been used as the gold standard in phytoextraction investigations for decades due to its significant metal affinity^8,32^. However, they are poorly biodegradable and unstable at high temperatures^33^. As a result, there have been concerns about HM leaching and associated ecotoxicity^34^. These problems are driving the quest for biodegradable and natural alternatives that are as effective as or better than EDTA at chelating heavy metals.

In recent years, chelates like citric acid (CA) have been used extensively in phytoextraction studies to improve plant growth, metal bioavailability, absorption, and translocation of metals throughout the plant. It contributes to reducing environmental pollution and toxicity to plants, which eventually increases the effectiveness of phytoremediation^8,14,31^.

Citric acid has a lot of ability to chelate heavy metals in the medium and get absorbed by plant roots at a faster rate because of its small-sized molecules and is more readily biodegradable than EDTA^35^. Even in multi-metal environments, it demonstrates high biodegradability and complexation stability without increasing the risk of leaching^36^.

The objective of this study was to assess the phytoextraction potential of *Cucurbita pepo*, *Lagenaria siceraria*, and *Raphanus sativus* in multi-contaminated soil with moderate-heavy metal levels, along with the helpful role of citric acid in the stress-relieving and phytoextraction processes.

## Results

### Plant biomass

The interaction effect between the tested species and citric acid treatment had significant effects on the means of fresh and dry weights of roots and shoots, as well as the root-shoot ratio (Table 1). Great differences in biomass were observed between tested species. The application of citric acid further significantly increased the fresh and dry weights of roots and shoots as compared to plants without citric acid addition. *R. sativus* followed by *C. pepo* exhibited the highest values compared to *L. siceraria* grown in the contaminated soil with or without citric acid treatment. The application of citric acid significantly increased the root-shoot ratio values of *C. pepo* and *L. siceraria* plants but had no remarkable effect on the root-shoot ratio values of *R. sativus* plants.

**Table 1:** Effect of the interaction between citric acid treatment and the tested species on the fresh and dry weight of roots and shoots per plant and root-shoot ratio.

Treatments		Fresh weight (g)		Dry weight (g)		
Citric acid	Species	Root	Shoot	Root	Shoot	Root-shoot ratio
Without	*C. pepo*	3.110 d	20.738 b	0.560 e	2.620 d	0.214 b
	*L. siceraria*	3.687 b	16.527 c	0.517 f	2.310 e	0.224 a
	*R. sativus*	2.840 e	16.124 c	0.602 c	3.340 b	0.180 e
With	*C. pepo*	3.340 c	29.303 a	0.617 b	3.273 b	0.189 d
	*L. siceraria*	3.863 a	21.061 b	0.594 d	2.933 c	0.203 c
	*R. sativus*	3.253 c	21.988 b	0.715 a	3.960 a	0.181 e
LSD at 0.05		0.119	1.384	0.006	0.119	0.007

a, b, c,…: LSD (p < 0.05) groups, means followed by a common letter in the same column do not differ significantly.

### Heavy metal concentrations in plant

The concentrations of studied heavy metals in roots and shoots are presented in Tables 2 and 3. They were significantly affected by the interaction between the tested species and citric acid treatment. *R. sativus* roots and shoots exhibited the highest Cd, Co, Cr, Ni, Pb, and Zn concentrations compared to the other tested species under with or without citric acid treatment. The concentrations of Cu in *C. pepo* roots and shoots were significantly higher than *R. sativus* roots and shoots of plants grown under with or without citric acid treatment. The addition of citric acid to the contaminated soil significantly increased the Cd concentration in the root and shoot of the plants. The results also indicated that the concentrations of heavy metals in the roots and shoots can be arranged in this order: Zn, Cr, Cu, Ni, Pb, Co, and Cd.

**Table 2:** Effect of the interaction between citric acid treatment and the tested species on the concentrations of studied heavy metals in roots (mg kg^**-**1^ dw).

Treatments		Cd	Co	Cr	Cu	Ni	Pb	Zn
Citric acid	Species							
Without	*C. pepo*	4.13 e	14.35 e	119.56 e	52.35 d	47.18 e	19.62 f	181.88 f
	*L. siceraria*	4.01 f	16.57 d	115.94 f	49.95 e	41.57 f	22.93 e	193.12 e
	*R. sativus*	5.62 b	18.46 c	121.37 d	48.38 f	50.81 c	25.93 c	196.86 d
With	*C. pepo*	4.92 c	18.28 c	160.79 b	62.58 a	56.82 b	24.98 d	199.48 c
	*L. siceraria*	4.80 d	19.98 b	152.63 c	59.16 b	49.76 d	27.52 b	205.91 b
	*R. sativus*	5.97 a	22.95 a	164.02 a	55.24 c	63.55 a	32.87 a	214.88 a
LSD at 0.05		0.088	0.586	0.498	0.411	0.815	0.110	2.495

a, b, c,…: LSD (p < 0.05) groups, means followed by a common letter in the same column do not differ significantly.

**Table 3:** Effect of the interaction between citric acid treatment and the tested species on the concentrations of studied heavy metals in shoots (mg kg^**-**1^ dw).

Treatments		Cd	Co	Cr	Cu	Ni	Pb	Zn
Citric acid	Species							
Without	*C. pepo*	2.23 f	7.56 f	66.47 e	42.15 e	35.65 d	7.52 f	124.58 e
	*L. siceraria*	2.43 e	8.25 e	64.86 f	44.69 d	36.83 d	9.68 e	131.55 d
	*R. sativus*	2.86 b	8.73 d	69.27 d	38.46 f	38.15 c	10.19 d	134.79 d
With	*C. pepo*	2.66 d	11.04 c	86.77 b	51.25 b	41.42 b	19.11 c	146.39 c
	*L. siceraria*	2.80 c	11.79 b	83.67 c	52.70 a	42.00 b	20.86 b	151.37 b
	*R. sativus*	3.21 a	12.63 a	89.16 a	48.12 c	43.51 a	21.64 a	159.95 a
LSD at 0.05		0.007	0.051	0.114	0.827	1.230	0.039	4.376

a, b, c,…: LSD (p < 0.05) groups, means followed by a common letter in the same column do not differ significantly.

### Bioconcentration factor (BCF)

The interaction effect between the tested species and citric acid treatment had significant effects on the bioconcentration factor (BCF) for all studied metals (Table 4). The BCF values of *R. sativus* were significantly higher (P < 0.05) than two other plant species for all studied metals except Cu under or without citric acid treatment. *C. pepo* plants showed the highest BCF for Cu followed by *L. siceraria* and *R. sativus* under with or without citric acid treatment. Generally, the citric acid application produced the maximum values of BCF. The values of BCF for the studied metals follow the order of: Cd > Ni > Cr > Zn > Cu > Co > Pb. The BCF values were above 1 for Cd in *R. sativus* with and without citric acid application and for Ni in *R. sativus* with the application of citric acid. The BCF values for Co and Pb were lower than 0.5 for all three tested plants, with the lowest BCF for Pb (0.196) in *C. pepo* without the application of citric acid.

**Table 4:** Effect of the interaction between citric acid treatment and the tested species on bioconcentration factor (BCF).

Treatments		Cd	Co	Cr	Cu	Ni	Pb	Zn
Citric acid	Species							
Without	*C. pepo*	0.826 e	0.287 e	0.598 e	0.523 d	0.786 e	0.196 e	0.606 f
	*L. siceraria*	0.801 f	0.331 d	0.580 f	0.499 e	0.693 f	0.229 b	0.644 e
	*R. sativus*	1.124 b	0.369 c	0.607 d	0.484 f	0.847 c	0.259 c	0.656 d
With	*C. pepo*	0.983 c	0.366 c	0.804 b	0.626 a	0.947 b	0.250 d	0.665 c
	*L. siceraria*	0.960 d	0.400 b	0.763 c	0.592 b	0.829 d	0.275 b	0.686 b
	*R. sativus*	1.194 a	0.459 a	0.820 a	0.552 c	1.059 a	0.329 a	0.716 a
LSD at 0.05		0.018	0.012	0.003	0.004	0.013	0.001	0.008

a, b, c,…: LSD (p < 0.05) groups, means followed by a common letter in the same column do not differ significantly.

### Translocation factor (TF)

Table 5 shows the TF values of studied metals for the three tested plants grown in the contaminated soil with or without citric acid treatment. The TF values of studied metals varied by plant species and citric acid treatment, and they were generally < 1.0. *L. siceraria* plants had the highest TF values of most studied metals compared to the other tested plants. The TF values of Co, Cu, Pb, and Zn for all tested plants were enhanced by the application of citric acid, while the TF values of Cd, Cr, and Ni were decreased by the application of citric acid.

**Table 5:** Effect of the interaction between citric acid treatment and the tested species on translocation factor (TF).

Treatments		Cd	Co	Cr	Cu	Ni	Pb	Zn
Citric acid	Species							
Without	*C. pepo*	0.540 c	0.527 c	0.556 c	0.805 cd	0.756 c	0.383 d	0.685 b
	*L. siceraria*	0.607 a	0.498 d	0.559 b	0.895 a	0.886 a	0.422 c	0.681 b
	*R. sativus*	0.508 d	0.473 e	0.571 a	0.795 d	0.751 c	0.393 d	0.685 b
With	*C. pepo*	0.540 c	0.604 a	0.540 f	0.819 c	0.729 d	0.765 a	0.734 a
	*L. siceraria*	0.582 b	0.590 a	0.548 d	0.891 a	0.844 b	0.758 a	0.735 a
	*R. sativus*	0.538 c	0.550 b	0.544 e	0.871 b	0.685 e	0.658 b	0.744 a
LSD at 0.05		0.012	0.018	0.002	0.015	0.017	0.023	0.017

a, b, c,…: LSD (p < 0.05) groups, means followed by a common letter in the same column do not differ significantly.

### Removal metal percentage (R%)

**Figure 1:**
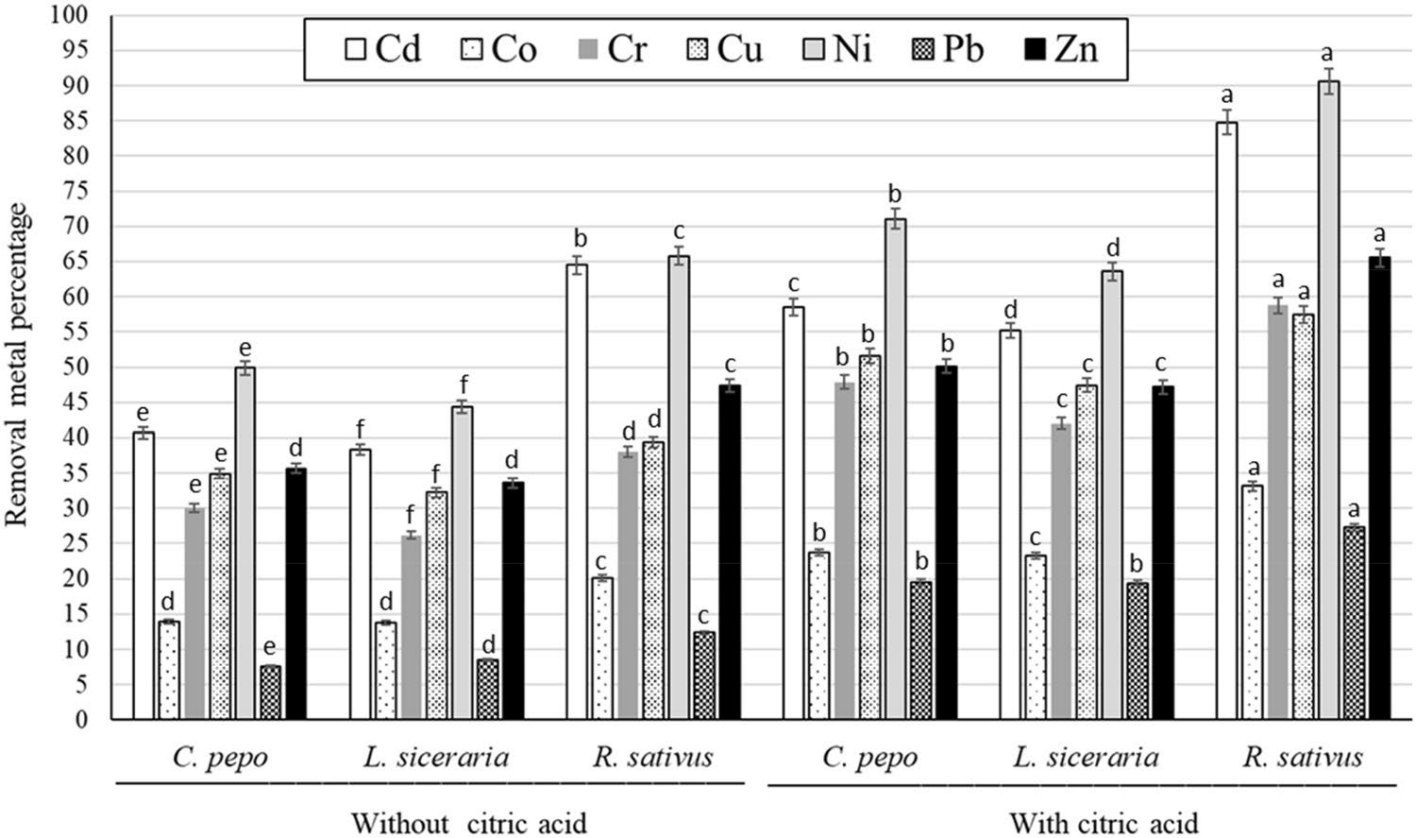
Effect of the interaction between citric acid treatment and the tested species on the removal metal percentage. Error bars are the standard deviation (different letters above bars represent significant differences at p < 0.05 among various treatments.

The R% of *C. pepo*, *L. siceraria*, and *R. sativus* for the studied metals under citric acid treatments is presented in Fig. 1. The interaction treatments had significant effects on the R%. Great differences were observed between different plants. *R. sativus* had the highest values compared to the other two tested plants with or without citric acid treatment. The application of citric acid significantly increased the R% of the three tested plants as compared to those without citric acid application. The R% of *R. sativus* for Cd, Co, Cr, Cu, Ni, Pb, and Zn were 84.80%, 33.17%, 58.75%, 57.48%, 90.64%, 27.28%, and 65.56%, respectively.

## Discussion

Results of this study show that the *R. sativus* plant produced the highest dry biomass compared to the other two plants within the relatively short frame of this experiment. Furthermore, applying citric acid to the soil significantly increased the growth and biomass production in all tested species, demonstrating the effectiveness of CA as a growth-promoting agent with chelating capability for various heavy metals^27,37^. Many studies documented the plant growth-promoting role of CA under the conditions of heavy metal-contaminated soil^38,39^. It is because that CA can improve chlorophyll content and photosynthesis efficiency in plant leaves^27,39^. Moreover, CA application can decrease the phytotoxicity of heavy metals by enhancing antioxidant enzyme activity^27,39,40^. This might be due to the function of CA to transform more toxic metals into less toxic forms^37^.

CA often enhance the bioavailability of metals and increase their accumulation by the plants which may cause a reduction in plant biomass due to the toxicity of extra uptake^40,41^. Therefore, the co-existence of CA is the best option to resolve this problem, which can also make the inherent antioxidative defense mechanism of a plant more efficient^40,42^. On the other hand, studies have shown that citric acid also plays an important role in increasing soil nutrient availability and uptake by the formation of complexes with nutrients, especially in calcareous soils^43,44^. With respect to the root-shoot ratio, it was observed that among the three tested species, *R. sativus* had the lowest values. While CA application minutely decreased the root-shoot ratio in *C. pepo* and *L. siceraria* plants, there was no discernible decrease in *R. sativus* plants. The root-shoot ratio is a very good indicator of the stress that plants suffer from heavy metals. According to Acuña et al.^45^, when plants are stressed by heavy metals, the root-shoot ratio increases.

The results of this study demonstrated that the levels of heavy metals in plant tissues varied depending on the species. *R. sativus* exhibited the highest values of metal concentrations compared to the other tested species. The success of phytoextraction is essentially dependent on a variety of variables, some of which are unique traits of plants, soils, or metals. However, plants' capacity to quickly produce huge amounts of biomass and their high efficiency of metal accumulation in shoot biomass largely determine the amount of metals they can extract^10,46^. Generally, plants should be harvested when the rate of metal accumulation in plants declines. This occurs at the end of the vegetative growth period. This will maximize the amount of heavy metals removed as well as minimize the duration of each growth cycle and allow more crops to be obtained in a growing season. The summer squash plants used in this study reach the end of their vegetative stage after 30 days from sowing.

The tissues of the plants' roots had a relatively higher concentration of metals than those of the leaves. The addition of CA further raised the levels of heavy metals in all plant parts. Similar observations on the enhancement of metal uptake by the application of citric acid under metal-contaminated soil have been reported on *Brassica juncea*^47^, *Kocuria rhizophiliai*^38^, *Brassica juncea*^8^, and *Brassica rapa*^8^. Also, higher metal concentrations in the roots than in the shoots were observed^8,38,47^. The enhancement of heavy metal uptake and translocation by citric acid might be related to the promotion of acid-soluble metal in the rhizosphere. The uptake of heavy metals by plants is closely associated with the root access rate and bioavailability of these metal ions. Citric acid acts as a solubilizing agent in the soil to increase the phytoavailability of heavy metals by desorbing them from the soil or by causing rhizosphere acidosis^40,48^. Furthermore, citric acid's chelating effect aids in the transfer of these metals from the roots to aerial plant parts^49^. Enhanced plant growth and biomass along with higher concentrations of heavy metals without any toxicity symptoms suggested that *R. sativus* is resistant to toxic levels of heavy metals. This brought to light the potential value of applying CA to phytoremediation techniques involving *R. sativus* plants.

Typically, the removal of heavy metals from soils and their accumulation in the plant need heavy metals bioaccumulation and translocation to parts of plants that may be easily gathered. The BCF and TF values were calculated (Tables 4 and 5) to assess whether plants were suitable for phytoremediation (i.e., phytoextraction or phytostabilization). The BCF and TF express a plant's capacity to take up metals from soils to roots and transfer those metals from roots to shoots, respectively (Zhuang et al.^50^; Padmavathiamma and Li^51^. Plants exhibiting BCF and TF values greater than one are suitable for phytoextraction, while plants with BCF values greater than one and TF values less than 1 are suggested to be more suitable candidates to remove metals from the soil through phytostabilziation (immobilization). However, plants with BCF and TF values less than 1 are not suitable for phytoextraction and phytostabilzation^52^. The highest BCF values among the heavy metals were found for Cd, whereas the lowest BCF values were found for Pb. A low BCF indicates that metals are strongly sorbing to colloids, whereas a high BCF indicates that metals are both relatively poorly retention and more mobile in the soil and therefore more effectively absorbed by plant roots^53^. The highest BCF values for Cd were largely consistent with the findings of the prior study^42^. The relatively decreased transfer of Pb from soil to plant roots shown by the lower BCF values is supported by the results of Hasan et al.^42^ and Acuña et al.^45^. Among the studied species, *R. sativus* had BCF values > 1 for Cd in the absence and application of CA and had BCF values > 1 for Ni in the application of CA. The higher value of BCF of Cd for *R. sativus* was strongly consistent with the findings of Hedayatzadeh et al.^20^ and Bortoloti and Baron^17^. The higher BCF values might be attributed to the concentration of metal available in the soil^54^. Results indicate the increased ability of plants to uptake heavy metals from the soil when CA was applied. Due to CA's ability, it may have lowered pH and secreted potent ligands in the soil, increasing Cd's solubility and bioavailability, and facilitating heavy metal accumulation in roots^40^. It was found in numerous earlier research that CA application increases the amount of heavy metals that *Brassicaceae* plants can phytoextract^8,40,41^.

According to the findings, all heavy metals had TF values that were less than 1 for all treatments. These results show that only a little amount of the heavy metal was transported to the shoots and that the majority was kept in the roots. This indicated that this plant species did not have a phytoremediation capability because it showed a reduced ability to translocate heavy metals from the root to shoot tissues. This result was in contrast with the previous studies^18–20^, which reported that *R. sativus* is a hyperaccumulator species for heavy metals. This may be due to the potential of metal accumulator plants for the extraction of metal from soil occurring up to a certain level of concentration, after that when the concentration of metal increased the phytoextraction rate of metal or bioaccumulation coefficient was decreased^19^. Patel and Patra^55^ also found that the translocation factor decreases with the increase in the total metal concentration of soil. In the present study, the maximum allowable concentrations of heavy metals for agricultural soils were applied, and plants were under several heavy metals, which produce both antagonistic and synergistic effects. Moreover, Saleem et al.^56^ found that Cu was significantly accumulated in the roots at an earlier stage of growth and significantly transmitted to the shoots at a later stage of growth due to the iron plaque formation in the roots. This experiment was designed just for 30 days to conclude the results. Hence, there was a significant accumulation of heavy metals in the roots, and all TF values are below 1. Similar observations have been observed under Pb stress^45^. In accordance with previous findings, the application of citric acid helps in the translocation of these heavy metals from the roots to shoots^49^ by its metal-complexation ability and by increasing metal transport through the xylem, and by enhancing metal storage capacity in the shoots^47^. Thus, it can be inferred from the presented results that the CA application played the most effective role in inducing metal tolerance and reducing metal stress resulting in increased bioaccumulation and translocation efficiency of plants under heavy metal-contaminated soil. The BCF and TF values show that *R. sativus* is not likely to be a high-efficiency plant for Cd and Ni translocation from the root to the shoot, but it is appropriate for phytostabilizing them. In this regard, Hedayatzadeh et al.^20^ also reported that the roots of *R. sativus* as excluders were able to limit the transportation of metals to the aerial parts.

The metal phytoextraction capability of studied species was also evident by the removal of metal percentages. The content of root exudates, the type of plant, the bioavailability of pollutants in the soil, and other factors may all have an impact on that. The highest R% values were found for Cd followed by Ni in *R. sativus* plants. Applying CA significantly increased R% values for all studied species. The capacity of a specific plant species to remove heavy metals from soil depends on both its capacity to quickly produce large amounts of biomass and effectively its capacity to absorb and accumulate metals.

Overall, the findings showed that *R. sativus* is a phytoremediator plant with a good ability to remove metals from the soil, especially with the soil application of citric acid. Pollution of plants is frequently caused by phytoremediation. As a result, the plants turn toxic and must be treated to prevent other environmental damage. Contaminated biomass of phytoremediation can be disposed of and used in various ways, including direct disposal, liquid extraction, incineration, compaction, composting, and ashing^17^. On the other hand, citric acid plays an important role in enhancing plant growth and increasing soil nutrient availability and uptake, especially in calcareous soils^43,44^. But, in the broader context of food safety and security, it must not be applied in the fields of summer squash, bottle gourd, and radish production when their soils have moderate levels of heavy metals. Moreover, these studied plants are important edible crops over the world, so regulations for regular monitoring of these plants are recommended to ensure food safety and raise awareness of metal-contaminated vegetables, and prevent health problems brought on by consuming contaminated vegetables.

## Conclusion

In conclusion, the current results highlighted that *R. sativus* could extract Cd and Ni from metal-contaminated soil with high efficiency compared to the other investigated species. Results also indicate that citric acid application may be a safer option for increasing heavy metal uptake by *R. sativus* and by reducing heavy metal toxicity and enhancing biomass production. These results should be confirmed in real field conditions and over longer time periods by further studies. On the other hand, considerably more attention should be given to evaluating the heavy metal content in plants of summer squash, bottle gourds, and radish when adding citric acid to their soil through fertigation systems during their growing season as vegetable crops. However, more studies are needed to validate the findings of this study on a large scale under various environmental conditions and in real-field conditions. Moreover, further research is still required to fully comprehend the factors that affect plant-assisted phytoremediation processes, such as the types and concentrations of heavy metals, soil properties, and the addition of chelating agents and soil amendments.

## Materials and methods

### Soil collection and plant materials

The soil for this experiment was taken from the top layer (0–20 cm) of cultivated land in Mansoura, Dakahlia Governorate, Egypt (31° 25′ 16.1" E, 31° 03′ 07.8" N). The collected soils were air-dried, crushed, and passed through a 1-cm sieve before use. The physical and chemical parameters of the collected soil were determined by the standard procedure described by Ryan et al.^57^, as shown in Table 6. The tested plants in this study were summer squash (*Cucurbita pepo* L.) cv. Rivera F1, bottle gourd (*Lagenaria siceraria*) cv. local variety and radish (*Raphanus sativus* L.) cv. Balady. Seeds of summer squash were obtained from Samtrade-Unifert Misr company, Egypt, while, seeds of bottle gourd and radish were obtained from Horticulture Research Institute, ARC, Egypt. These vegetables were selected for this study due to their high germination rates, quick growth rates, high biomass production, and abiotic tolerance, which have all been shown to effectively remove heavy metals through phytoextraction. They were also chosen because they are locally and frequently produced in Egypt.

**Table 6:** Selected soil physical–chemical properties of the soil before treatments.

Parameter	Value
Clay (%)	39.68
Silt (%)	37.73
Sand (%)	23.59
Texture	Clay-loam
Organic matter (%)	1.78
EC (dS m^–1^) (1:10)	2.14
pH	7.91
Total Cd (mg kg^−1^)	2.35
Total Co (mg kg^−1^)	15.13
Total Cr (mg kg^−1^)	113.76
Total Cu (mg kg^−1^)	16.59
Total Ni (mg kg^−1^)	22.86
Total Pb (mg kg^−1^)	29.33
Total Zn (mg kg^−1^)	91.54

### Experimental setup

The pot experiment was undertaken outdoors at Mansoura Horticulture Research Station in Dakahlia Governorate, Egypt, from 11th April to 23rd May during the summer season of 2021. During the experiment, the monthly temperature ranged from 17.2 to 23.1 °C, with monthly rainfall of 70.97 mm, and the average relative humidity was 64.3%.

Air-dried soil was placed into plastic pots with a capacity of 7 kg. Pots were arranged in a randomized factorial design with two factors (2 × 3) including two citric acid levels (0 and 0.98 g kg^−1^ soil) and three plant species. There were four replicates of each treatment, with four pots per plot. All pots were treated with a suitable amount of heavy metal solution to achieve the artificially polluted soil. The heavy metal solution used in spiking was prepared from the following heavy metal salts: CdSO_4_.8H_2_O, CoCl_2_.6H_2_O, Cr_2_(SO_4_)_3_, CuSO_4_5H_2_O, Ni-SO_4_6H_2_O, Pb(C_2_H_3_O_2_)_2_ and ZnSO_4_7H_2_O. The final total element content in the soil was 5 mg kg^−1^ Cd, 50 mg kg^−1^ Co, 200 mg kg^−1^ Cr, 100 mg kg^−1^ Cu, 60 mg kg^−1^ Ni, 100 mg kg^−1^ Pb, and 300 mg kg^−1^ Zn, which were within their maximum allowable concentration for agricultural soils mentioned by Kabata-Pendias^58^. The heavy metal solution was slowly applied to avoid overflow from the pots. Pots were left for a few days after solution application, and then an equal amount of water was applied to ensure uniform contamination of heavy metals in the soil in the pots. For three weeks, the pots were left to equilibrate. During this period, tap water was added every 48 h to keep the soil moisture content at approximately 65 percent of the field capacity. At the end of the third week, citric acid solution, prepared in distilled water, was applied to half of the pots at the concentration of 0.98 g kg^−1^ soil.

Six seeds of three plant species were sown in each pot at 2 cm deep on 11 April 2021 and irrigated with tap water. The seedlings were thinned to two plants in each pot after two weeks of emergence. The plants that were taken out of the pot were carefully crushed and put back into the same pot. The pots were irrigated to maintain soil moisture at the field capacity level during the growth period of the experiment. After 20 days of sowing, all the pots were irrigated with NPK fertilizer solution at a rate of 90–45–30 kg per hectare, respectively.

### Sampling and measurements

After 30 days of sowing, the plants were gathered and carefully removed from the soil. All plants were carefully washed with tap water followed by deionized water, and the surplus water was absorbed using a napkin. Each plant's roots and shoots were carefully separated, and their fresh weights were recorded. Samples were then dried at 70 °C for 72 h to determine dry weights, ground into a fine powder, and kept in paper bags for heavy metal analysis. Samples (1g) of plant parts and soil were digested using a 4:1 mixture of nitric acid (HNO_3_) and perchloric acid (HClO_4_) in Teflon bottles. The samples were then heated for one hour at 40 °C and then increased to 170 °C for four hours until a clear solution was seen. The acidic solution was purified using Whatman No. 1 filter paper and then deionized water was used to dilute the acidic solution to a final volume of 50 ml. The concentrations of heavy metals in plant parts were measured using a Thermo Scientific TM ICAPTM 7000 Plus Series ICP-OES^59^. A certified reference material (CRM 1570) was used in accordance with the National Institute of Standards and Technology Standard to verify that the estimation was accurate. To maintain the quality of the dealings, triplicate analyzed samples were laid out, and the findings showed that the estimated trace metals were evaluated with a recovery level of 98.2%.

The bio-concentration factor (BCF), translocation factor (TF), and total heavy metal uptake in shoot and root parts, as well as the percentage of removal of metal (R%) were measured for each heavy metal according to Zhuang et al.^50^, Padmavathiamma and Li^51^, to evaluate the phytoextraction capacity of each plant species to accumulate heavy metals in their biomass.

The ratio of the metal concentration in harvested plant tissues to the soil is represented by the BCF. The ratio of the metal concentration in the plant shoot to root is known as TF. The TU was calculated by multiplying the biomass of each plant organ (shoot and roots) by its metal concentration. R % is the percentage of the ratio of the total metal accumulation in plants per pot to the initial metal amount per pot.

### Statistical analysis

The data were tabulated and analyzed using Microsoft Excel® 2013 and the Number Cruncher Statistical System statistical program. Also, statistical analyses were calculated using a 2-way analysis of variance (ANOVA) according to Snedecor and Cochran^60^ and means of treatments were checked for the significant difference using the Least Significant Difference (LSD) test at p < 0.05 in Costat computer software. Prior to ANOVA, Kolmogorov–Smirnov and Shapiro–Wilk tests were used to determine the homogeneity of variance and normal distribution of the data.

### Research involving plants statement

This study was developed with commercial seeds, therefore nonexotic or at risk of extinction, under controlled conditions, meeting all institutional, national, and international guidelines and legislation for cultivated plants.

## Data Availability

All data generated and/ or analyzed during this study are available from the corresponding author on reasonable request.
